# Tumor-Derived Extracellular Vesicles: Multifunctional Entities in the Tumor Microenvironment

**DOI:** 10.1146/annurev-pathmechdis-031521-022116

**Published:** 2022-10-06

**Authors:** James W. Clancy, Crislyn D’Souza-Schorey

**Affiliations:** Department of Biological Sciences, University of Notre Dame, Notre Dame, Indiana, USA

**Keywords:** extracellular vesicles, exosomes, microvesicles, EV cargoes, carcinoma-associated fibroblasts, tumor-infiltrating cells, cell migration, tumor invasion, liquid biopsies

## Abstract

Tumor cells release extracellular vesicles (EVs) that can function as mediators of intercellular communication in the tumor microenvironment. EVs contain a host of bioactive cargo, including membrane, cytosolic, and nuclear proteins, in addition to noncoding RNAs, other RNA types, and double-stranded DNA fragments. These shed vesicles may deposit paracrine information and can also be taken up by stromal cells, causing the recipient cells to undergo phenotypic changes that profoundly impact diverse facets of cancer progression. For example, this unique form of cellular cross talk helps condition the premetastatic niche, facilitates evasion of the immune response, and promotes invasive and metastatic activity. These findings, coupled with those demonstrating that the number and content of EVs produced by tumors can vary depending on their tumor of origin, disease stage, or response to therapy, have raised the exciting possibility that EVs can be used for risk stratification, diagnostic, and even prognostic purposes. We summarize recent developments and the current knowledge of EV cargoes, their impact on disease progression, and implementation of EV-based liquid biopsies as tumor biomarkers.

## INTRODUCTION

More than five decades ago, there were reports that cells in culture released small sacs of then unknown function (1). These vesicular structures, now known as extracellular vesicles (EVs), encompass a burgeoning field of biological research that could change our current understanding of cell communication while also holding great promise for disease diagnostics and therapeutics. It is now appreciated that all cells release EVs as a normal physiological process that appears to be usurped under pathophysiological states and especially during cancer progression (2, 3). Currently, EVs comprise a large number of unique particles, including exosomes, other small exosome-sized EVs, microvesicles (MVs), arrestin domain-containing protein 1-mediated microvesicles (ARMMs), apoptotic bodies, and large oncosomes (LOs) (4-6) (Table 1). The best known EV categories are microvesicles, oncosomes, and exosomes. Microvesicles and oncosomes pinch off the cell surface via the outward budding and fission of the plasma membrane, and they range in size from 200 nm to >1 μm in diameter (7). Exosomes, which range in size from ~60 to 100 nm in diameter, are formed within multivesicular bodies (MVBs) and are released into the extracellular space by fusion of the MVB limiting membrane with the cell surface (8-10). The term “extracellular vesicles” refers collectively to the heterogeneous population of membrane vesicles of cellular origin that derive from endosomes and, also, direct release from the plasma membrane. More recently, reports have described the identification of two unique extracellular particles (EPs), referred to as exomeres and supermeres, also released from cells (11, 12). These nanoparticles, which may be grouped along with supramolecular attack particles and chromatin particles (Table 1), lack an encompassing membrane but contain unique signatures of bioactive components, including protein, nucleic acid, lipid, and *N*-glycosylation markers, which together suggest distinct biological functions (13).

It is worth noting that the field is still evaluating what properties define individual EV and EP types and how to best isolate each class of EVs. As a result, many studies claiming to specifically study either exosomes or other EV types are in fact reporting conclusions based upon a mixture of EVs. This has prompted the EV community to adopt new guidelines, which include using the term “EVs” in cases where it is not absolutely clear that a particular class of EVs is being isolated and studied (14). Moreover, it is becoming increasingly evident that several subtypes of exosomes and microvesicles likely exist. For the purposes of this review, we have used the terminologies chosen by the authors when describing their work or the term “EVs” when undefined. Ongoing technological and experimental advances are likely to yield valuable information regarding EV heterogeneity and biological function. As more purification and analytical procedures for the study of EVs are developed, additional information about their functional heterogeneity will come to light. Nonetheless, functional readouts using mixed pools of EVs or those enriched for specific populations have provided new insights into their contribution to various diseases, most notably in cancer progression (15). Indeed, EVs have been implicated in several aspects of tumor progression including tumor growth and invasion, drug resistance, and metastatic dissemination (2, 3).

Depending on the cell of origin, EVs may contain various types of cellular cargoes including DNA, various RNA species, lipids, metabolites, signaling molecules, and cell-surface receptors that may be taken up by other cells, both in the direct vicinity of the source cell and at distant sites in the body following transmission in biofluids (4, 16). This ability to transfer cargo and elicit a variety of phenotypic responses has been the focus of intense investigation, though the roles of EV subtypes in the removal of excess and/or unnecessary constituents from cells have been recently revisited (17, 18). Because of their distinctive biology and roles in cell–cell communication, the study of EVs has attracted strong interest, which is further enhanced by their potential clinical utility. EVs have been reported in almost all biological fluids, where they are readily sampled for clinical analysis (liquid biopsies). Indeed EV-based liquid biopsy highlights their potential functionality in diagnosis and determining the prognosis of patients with cancer and other diseases (19, 20). Disease progression and response to therapy may also be ascertained by analysis of EVs. Moreover, EVs can be engineered to deliver therapeutic payloads, including short hairpin RNAs, chemotherapeutic agents, cytokines, and other immune modulators to target cells of interest (21-23). In this regard, the composition of EVs lends to their enhanced bioavailability and to minimizing adverse effects while in circulation.

Over the last decade, the EV field has flourished, with many new reports touching nearly every field of study under the biomedical umbrella. One of the challenges before the EV research community is to develop an understanding of EV biology as an ecosystem—including a renewed focus on the cell biology of biogenesis and secretion of individual EV subtypes, the mechanisms of cargo exchange, and the signaling pathways that allow cells to communicate with other cells and their microenvironment. Here, we review studies that point to a functional role for EVs in regulating intercellular communication in the tumor microenvironment. The multitude of bioactive EV cargoes delivered to stromal cells effectively induces a biological response in recipient cells. These responses help tumor cells to evade antitumor immunity, modify the tumor microenvironment, and create an environment that is conducive to tumor growth. The plethora of material contained within EVs hints at the enormous impacts stemming from these small packages of biological significance.

## EXTRACELLULAR VESICLES CONTAIN A DIVERSE ARRAY OF MULTIFUNCTIONAL CARGO

Owing in large part to the complex, multimodal, biogenesis pathways responsible for their formation and release, EVs contain a treasure trove of molecular cargo (6, 24). As of this writing, EV researchers have identified and characterized numerous components, and the field remains intensely focused on a more complete and more refined understanding of EV cargo. Broadly, tumor EVs are known to contain most classes of bioactive molecules, including proteins, lipids, metabolites, genetic material, and organelle fragments, and often contain a rich assortment of each subtype.

### Extracellular Vesicles Contain Distinct Proteomic Signatures

A large number of studies have been conducted to investigate the protein composition of EVs, mainly by profiling the contents of different-sized EVs produced by various cell types. However, due to the different cell types used, as well as variations in isolation techniques, one has to be cautious about drawing a conclusive picture of the protein composition of the EV subtypes. As the sections below describe, what is apparent, however, is that commonly found proteins in EV subtypes are those associated with biogenesis pathways, including proteins associated with the endomembrane system.

#### Tumor microvesicles are enriched with bioactive protein cargo.

For tumor-derived microvesicles (TMVs), shedding from the cell surface is the culmination of an intricately choreographed set of cell processes that simultaneously regulate the trafficking and delivery of specific molecular cargo together with activation of the intrinsic contractile machinery needed for pinching and release. Sites of active TMV release serve as convergence points allowing the integration of multiple intracellular trafficking pathways, many of which are regulated by signaling GTPases, which are subsequently included as TMV cargo. These include multiple members of the Ras-related GTPases including RhoA, Rab22A, Rab35, and ARF6 (25-29). Given the related regulatory roles for several of these same GTPases in directing intracellular endomembrane trafficking, it is no surprise that cargo known to traffic through those same pathways is frequently found as TMV cargo. For example, not only does the TMV regulator ARF6 regulate the phosphorylation and activation of the actomyosin contractile machinery, but also ARF6-positive endosomes serve as a repository for TMV cargo, including β_1_-integrin, major histocompatibility complex 1 (MHC-1), and membrane type-1 matrix metalloprotease (MT1-MMP or MMP14) (27). MT1-MMP, for example, traffics through ARF6 endosomes and, in a mechanism in part regulated by a CD9-dependent interaction with the v-SNARE vesicle associated membrane protein 3 (VAMP3), is delivered to the cell surface and incorporated as TMV cargo (30). Importantly, the VAMP3-dependent delivery of MT1-MMP is not limited to newly synthesized protease; ARF6-regulated endosomes similarly hold reserves of recycled protease, which can also be rapidly delivered to sites of nascent TMV biogenesis. Interestingly, not all cargo that moves through ARF6 endosomes is included as TMV cargo, highlighting the specificity and the critical role for trafficking regulators in determining the delivery and enrichment of certain molecules (27). Recently, a proteomics-based study of multiple EV subtypes reported some level of specificity for the acidic phospholipid binding protein annexin A1 as a marker of EVs, including TMVs, which are shed directly from the cell surface. While the distribution of annexin A1 among the plasma membrane-derived EV subtypes remains to be determined, subsequent work has confirmed its presence in isolated TMVs (31). Interestingly, this same proteomics study identified ARF6 in both large EV (LEV) and small EV (sEV) fractions, suggesting the potential of smaller sized (<200 nm) microvesicles (31, 32). In addition to the proteins listed above, multiple additional components have been detected within purified TMVs. These components include actin and the actin bundling protein fascin; lipid microdomain components including flotillin-1; multiple members of the CEA cell adhesion molecule (CEACAM) family including CEACAM1, CEACAM3, and CEACAM5; multiple putative tumor biomarkers including CA-125; RNA processing machinery including dicer and argonaute-2; and DNA binding proteins (25, 30, 33, 34). While TMVs contain abundant cargo, they contain virtually undetectable levels of proteins such as TSG-101, CD81, and CD63, separating them from other well-studied EVs including exosomes (6, 7, 24).

#### The exosome proteome.

Following the identification of members of the tetraspanin family, including CD9, CD37, CD63, CD81, and CD82, being highly enriched on intraluminal vesicles and shed exosomes, the field has frequently used these proteins as identifiers of exosome pools. Subsequent investigations built a large repertoire of additional protein components based on co-isolation, or immunoaffinity/immunocapture methods to specifically isolate the tetraspanin-positive membrane fractions (31, 35, 36). As tetraspanins lack intrinsic catalytic activities but instead facilitate the trafficking and oligomerization of other membrane proteins, it is no surprise that a large number of tetraspanin-associated proteins including integrins, syndecans, immunoglobulin, MHC isoforms, Rac GTPase, ADAM10, and ezrin-radixin-moesin proteins have been identified within exosome fractions (24, 31, 35, 37). As mentioned previously, research advancing our understanding of EV heterogeneity is leading to evolution of EV nomenclature and classification. This is most apparent within the field of exosome biology, where refinement of isolation techniques has resulted in the realignment and reclassification of the most well-studied EVs (11, 12, 31, 36). Several reports have demonstrated that sucrose gradient ultracentrifugation of isolated exosomes allows for the separation of multiple distinct subpopulations with differing protein content and profiles, including levels of the classical exosome markers CD81, CD9, and CD63 (36, 38). Interestingly, researchers have found that there are protein markers that are generic to sEVs including annexin XI, flotillins, ADAM10, and EHD4 whether or not they contain any of the classical tetraspanin markers. Other proteins, including members of the endosomal sorting complex required for transport (ESCRT) such as CHMP4A and TSG101, are isolated upon immunocapture of sEVs using the three tetraspanins listed above. Perhaps most intriguing are the proteins like the ESCRT family member ALIX that can be immunoprecipitated with CD9^+^ exosomes but are absent in the precipitates generated using CD81 or CD63 beads. The heterogeneity in cargo is only further exemplified when these results are combined with a similar study that identified high- and low-density exosome pools containing both ALIX and TSG101. Notably, these authors measured detectable levels of CD9, CD81, and CD63 in both pools. Together with these advances, several recent reports have also attempted to identify universal exosome markers as a means to distinguish between distinct EV subtypes (31, 35, 39).

#### ARMMing the system—the proteome of small extracellular vesicles shed from the cell surface.

Size and mechanism of biogenesis are among the defining characteristics that have separated the two most well-studied classes of EVs: exosomes and microvesicles. Residing at the intersection of these two intrinsic characteristics is a novel class of sEVs, the ARMMs, which form via an outward budding and pinching mechanism, like MVs do, but have an average diameter of approximately 45 μm, in line with exosomes (40). Initial characterization of the ARMM class of MVs centered around their ARRDC1-dependent mode of biogenesis, in which ARRDC1 recruits TSG-101, a component of the ESCRT-I complex, to initiate budding and release of ARMMs containing both proteins. In a follow-up report, the authors examined the proteomic cargo of ARMMs by liquid chromatography–tandem mass spectrometry, whereby researchers were able to identify 177 proteins that were enriched >1.5-fold in green fluorescent protein (GFP)-ARRDC1 ARMMs relative to vector controls and a further 65 proteins that were detected only in GFP-ARRDC1 vesicles and not in control vesicles. Among the proteomic cargo, the authors noted significant enrichment of TSG-101 and other components of the ESCRT complexes including CHMP1B, CHMP3, and CHMP6; FAM125A; VPS25, −28, and −36; and VPS37A, B, C, and D. In addition to the heavy enrichment of ESCRT machinery, the authors also identified proteasome components, members of the integrin family, and several NEDD4 E3 ligases including WWP1, WWP2, and ITCH (41). Interestingly, while the authors noted that several putative exosome markers (CD9 and CD81) did not markedly change between control and ARRDC1 vesicles, they also identified the TMV marker ARF6. It remains to be seen whether these proteins are significant ARMM cargo components or merely the result of some carryover of vesicle populations, given the partially overlapping size profiles. Finally, cargo trafficking to ARMMs appears to be at least partially dependent on ARRDC1, as engineering of fusion proteins to express ARRDC1 or interacting domains leads to their efficient loading and release as ARMM cargo. These proteins include ARDCC1 fused to the tumor-suppressor p53; combinations of ARDCC1 fused to the transactivator of transcription, which binds the transactivating response (TAR) element together with TAR-fused messenger RNAs (mRNAs); and WW domain–fused Cas9, which interacts with ARRDC1 through its PPxY motifs (42).

### Messages in a Bubble—Tumor-Derived Extracellular Vesicle Nucleic Acid Cargoes

In addition to protein cargo, there is extensive literature documenting the inclusion of multiple forms of nucleic acids as EV cargo. Within the TMV class, significant advances in our understanding of nucleic acid cargo and cargo trafficking have built the foundation for ongoing studies. These include multiple reports that sought to identify and characterize RNA cargo, in particular, microRNA (miRNA) cargo, within shed vesicles. In two reports, the movement of miRNA cargo was dependent on the interaction of the nucleic acid cargo [either miRNA or precursor (pre)-miRNA)] with chaperone proteins, which are themselves then included as TMV cargo. In the first study, oxidative stress led to the *O*-GlcNAcylation modification of hnRNPA2B1 and subsequent alterations to associated miRNA cargo (43). In the second study, pre-miRNA cargo was trafficked out of the nucleus together with the export chaperone exportin-5. This complex was then transferred to active, GTP-bound ARF6, facilitating the movement into nascent TMVs (33). In addition to miRNA, TMVs have been reported to contain numerous other forms of RNA cargo including mRNA, ribosomal RNA (rRNA), and transfer RNA (tRNA) (16, 33, 44). Beyond RNA, EVs have been reported to contain measurable quantities of double-stranded DNA (dsDNA). A pair of reports using prostate cancer models have highlighted the dsDNA content within the larger populations of EVs including large oncosomes. In the first study, following the purification of genomic DNA from large EVs, the authors subsequently conducted whole genome sequencing to reveal that genomic alterations could be identified within LO DNA (45). The second report examined the source of LO DNA, finding that nuclear instability resulted in an increase in LOs with dsDNA cargo (46). It was also recently reported that TMVs contain a pool of dsDNA that is protected from nuclease degradation. Following iodixanol gradient centrifugation procedures, dsDNA containing a plethora of genes can be isolated from multiple gradient fractions but is only membrane protected in fractions that also contain TMV markers (32). The authors went on to demonstrate that the inclusion of this dsDNA cargo is dependent upon the nucleotide binding state of the small GTPase ARF6, and that it traffics together with the canonical cytosolic DNA sensor cGAS. Moreover, dsDNA can then be transferred to recipient cells, where it leads to transcription and translation of the encoded genes and alterations to recipient cell behavior.

A complete understanding of nucleic acid cargo within shed exosomes remains an unsettled issue within the field. Numerous studies have reported that exosomes contain some quantity of membrane-enclosed nucleic acids that are protected from nuclease degradation without detergent disruption. Similar to reports on TMVs, these studies include reports of multiple species of DNA and a similar variety of RNAs including both coding (mRNA) and noncoding [YRNA, miRNA, mitochondrial RNA (mtRNA), small nuclear RNA (snRNA), long noncoding RNA (lncRNA), small nucleolar RNA, etc.] species capable of inducing a biological effect (16, 24, 47-51). More recently, however, it has been reported that the release of extracellular nucleic acids, which sediment with exosome membrane fractions, is not associated with exosomes themselves but rather is associated with extracellular protein/nucleic acid complexes, some of which facilitate the active release of extracellular chromatin via the same multivesicular bodies that are the source of exosomes (31). This would represent a remarkable shift in our understanding of exosomes and is certainly worthy of follow-up studies to investigate the roles of exosomal heterogeneity, tumor- or cell-type specificities, and the need for continued development of EV isolation and purification technologies.

As mentioned previously, the DNA content found within large oncosomes is reported to represent the mutational landscape of the shedding cells. A recent report highlighted a similar finding when researchers examined the exosomal DNA (exoDNA) content in the plasma of patients with neuroblastoma. Following treatment with deoxyribonuclease I, purified exoDNA was subjected to a whole-exome sequencing pipeline. Neuroblastoma exoDNA spanned the entirety of the exome, could be used to identify genetic variations conferring resistance (*TP53*, *RAS*, *ALK*), and carried tumor-specific mutations including multiple known neuroblastoma tumor suppressors and oncogenes (*ALK*, *SHANK2*, *FGFR1*, *BRAF*, etc.) (52). In a similar study, genome-wide methylation profiling of exosomes derived from glioblastoma demonstrated that the methylation profile of EV DNA matched that of the originating tumor and shedding cells. It is worth noting that these researchers examined both internal and external DNA and that in addition to the methylation profile, they were able to accurately detect copy-number variations and tumor-specific mutations (53).

## TUMOR EXTRACELLULAR VESICLES IN THE TUMOR MICROENVIRONMENT

**Figure 1:**
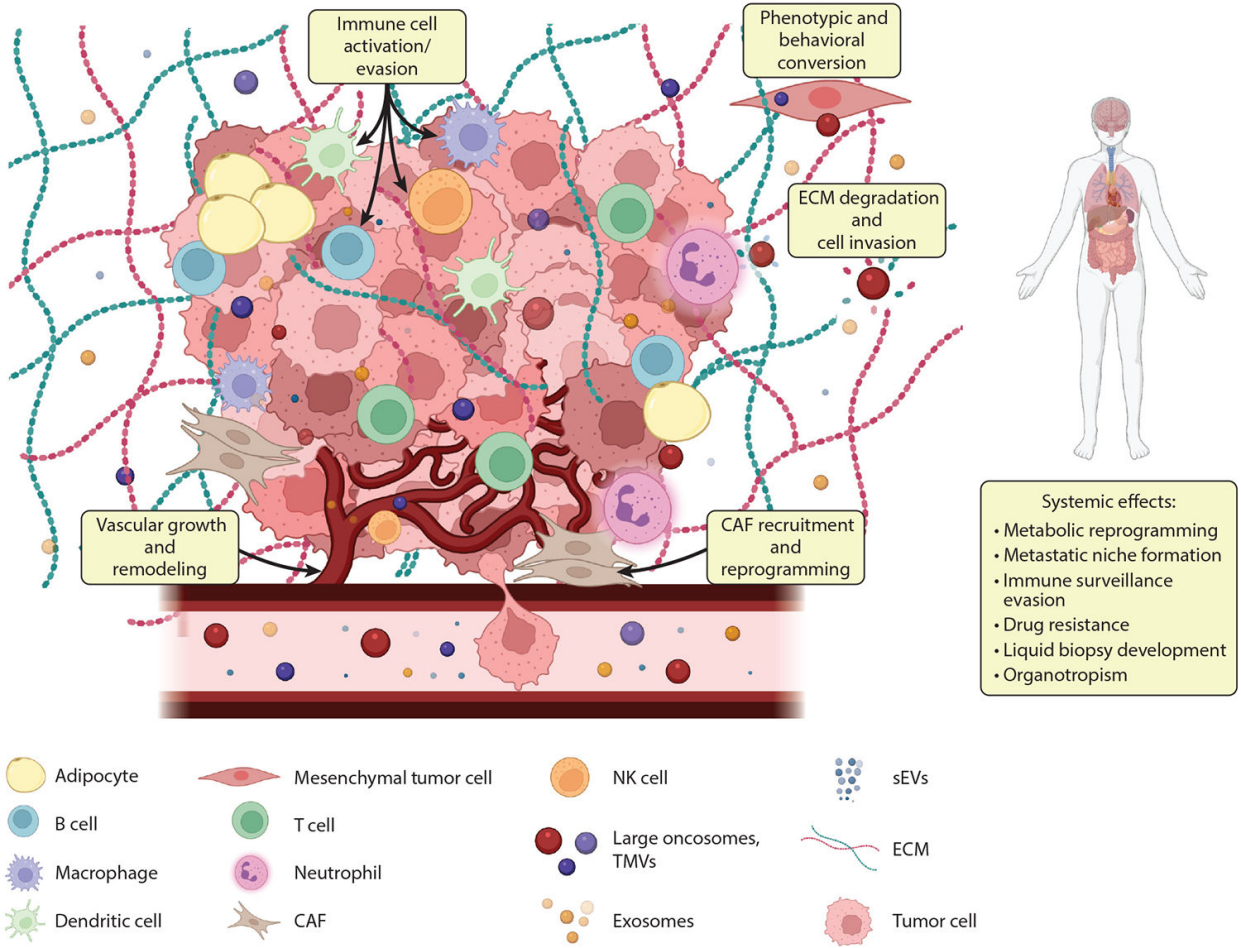
EVs in the TME. The TME encompasses diverse cell types that include heterogeneous cancer cells, immune cells, stromal cells, and other tissue-specific cell types; the blood and lymphatic vascular networks; the ECM; and secreted factors such as EVs. It is increasingly understood that EVs, which include large oncosomes, TMVs, sEVs, and exosomes, facilitate both bidirectional communication in the TME and matrix degradation, inducing both local and systemic effects. Circulating EVs represent promising platforms for biomarker development, as highlighted in this review. Abbreviations: CAF, cancer-associated fibroblast; ECM, extracellular matrix; EV, extracellular vesicle; NK, natural killer; sEV, small EV; TME, tumor microenvironment; TMV, tumor-derived microvesicle. Figure adapted from images created with BioRender.com.

**Figure 2:**
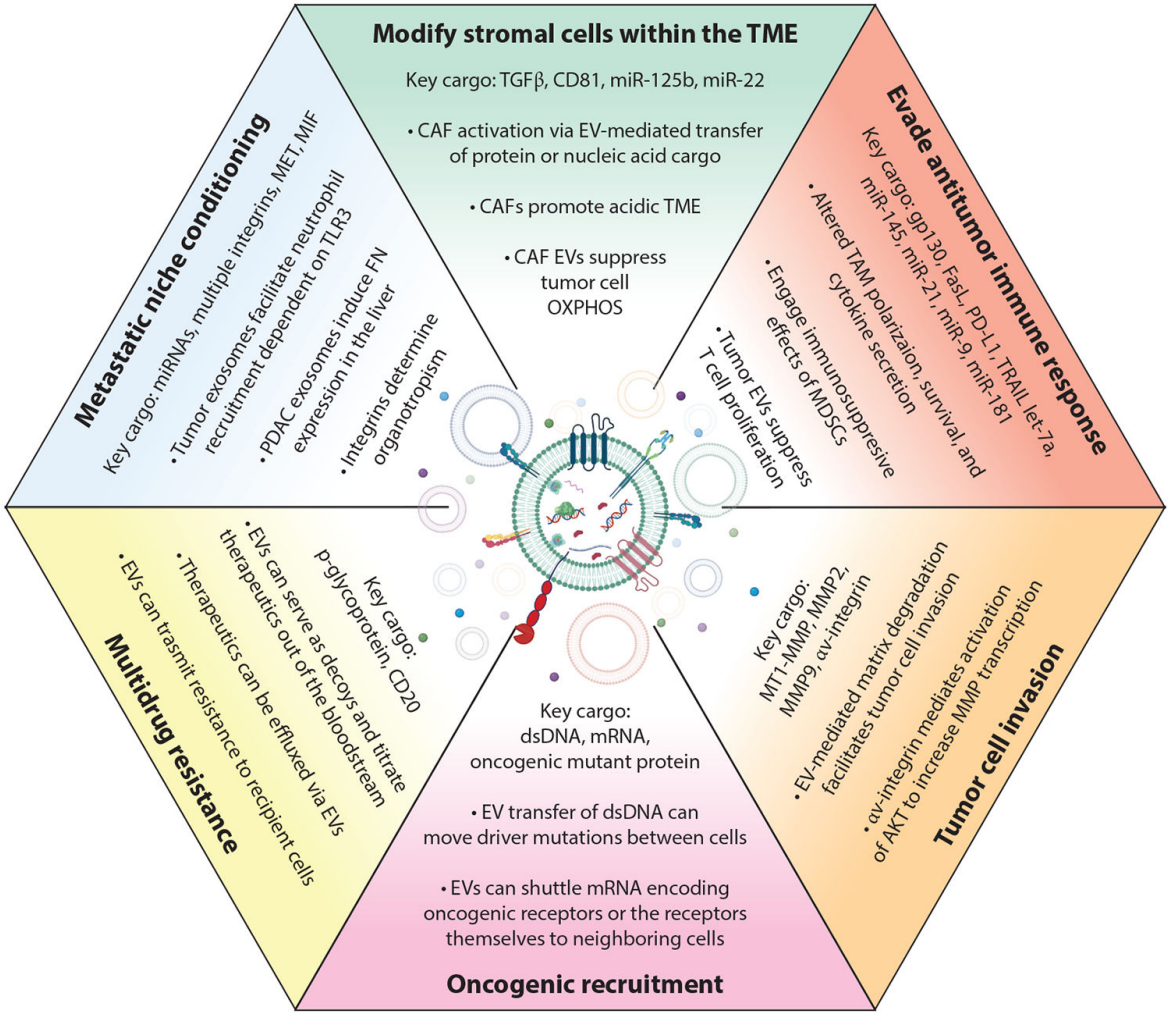
EVs have multiple documented roles within the TME. Due to the abundant bioactive cargo contained within EVs, they are capable of affecting multiple biological processes within the TME. Many of these effects, a selection of which are highlighted, can be mediated through both the direct action of EV cargo and/or the transfer of bioactive molecules to recipient cells. Abbreviations: CAF, cancer-associated fibroblast; dsDNA, double-stranded DNA; EV, extracellular vesicle; FN, fibronectin; MDSC, myeloid-derived suppressor cell; MMP, matrix metalloprotease; OXPHOS, oxidative phosphorylation; PDAC, pancreatic ductal adenocarcinoma; TAM, tumor-associated macrophage; TME, tumor microenvironment. Center image created with BioRender.com.

Tumors develop in a complex, dynamic, and interconnected microenvironment that influences multiple facets of their growth, development, invasion, and metastatic progression (Figure 1). This region, typically referred to as the tumor microenvironment (TME), is composed of a broad array of both cellular and noncellular components. These include numerous stromal cell types including multiple forms of tumor-infiltrating immune cells [infiltrating lymphocytes including myeloid-derived suppressor cells, B cells, T cells, and tumor-associated macrophages (TAMs)], tumor-associated endothelial cells, cancer-associated fibroblasts (CAFs), and tumor-associated adipocytes. In addition to the cellular components described above, the TME contains a multitude of noncellular components that form the extracellular matrix (ECM) including laminin, hyaluronan, collagen, and fibronectin (54). Throughout the development of the tumor, there exists strong, reciprocal communication between the tumor and the TME, including both cell–cell and cell–ECM interactions. It is increasingly understood that EVs are critically important actors within the TME, facilitating bidirectional communication between the malignant and nonmalignant components of the TME (Figure 2). Here, we focus on EV-mediated interactions with CAFs, immune cells, and the ECM.

### Tumor-Derived Extracellular Vesicles Facilitate Cross Talk with Carcinoma-Associated Fibroblasts

The most well studied of the EV subtypes with documented roles in the TME are exosomes, and research into their many functions during cancer has progressed in line with the rapid expansion of the EV field. EV-mediated stromal interactions are most commonly utilized in vitro to examine the effects of tumor-derived EVs on recipient fibroblasts, models for CAF communication, and/or transformation. Exosomes released from tumor cells, can, for example, promote neoplastic development and progression through the initiation of oncogenic transformation. Incubation with pancreatic tumor cell exosomes leads to the random incorporation of molecular changes within recipient NIH/3T3 fibroblast DNA, conferring tumor-forming capability when implanted into mice (55). Furthermore, signaling induced by the exosome-mediated delivery of transforming growth factor beta (TGFβ) led to SMAD-dependent fibroblast activation, including expression of αSMA and FGF2 (56). Utilizing an in vitro model for chemoresistant non-small cell lung cancer (NSCLC), researchers have also highlighted a role for tumor-derived exosomes in reprogramming CAFs to form an acidic TME. This environmental change subsequently promotes NSCLC proliferation and enhances resistance to platinum-based chemotherapeutics (57).

The EV-mediated communication between tumor cells and CAFs is not unidirectional. A growing body of evidence demonstrates that exosomes participate in cross talk between the tumor and the surrounding stroma with significant pathological implications ranging from metabolic reprogramming and response to hypoxic stress to cell migration and immune modulation. Depleting focal adhesion kinase in exosome-shedding CAFs reduced breast cancer metastasis in part through blocking the ability of CAF-derived exosomes to deliver functional miRNA cargo to tumor cells (58). Similarly, CAF-derived CD81^+^ exosomes lead to tumor cell activation and the initiation of a Wnt-planar cell polarity autocrine signaling axis, which supports breast cancer cell migration and invasion (59). CAF-derived exosomes have also been reported as a reservoir of mtDNA, which can be delivered to breast cancer cells. The resulting expression of mtRNA leads transduced cells to break dormancy in vivo, induces oxidative phosphorylation (OXPHOS), and promotes survival (60). Interestingly, prostate and pancreatic CAF-derived exosomes have been reported to suppress OXPHOS in recipient tumor cells, through the delivery of miRNA cargo including miR-125b, let-7a, and miR-22, which were among a group of miRNAs found in exosomes reported to target OXPHOS. These researchers went on to determine that the inhibitory effects on mitochondrial OXPHOS stemming from exosomal miRNA cargo were balanced by the EV-mediated transfer of intact metabolites including tricarboxylic acid–cycle intermediates and lipids, which were readily utilized by recipient tumor cells to promote tumor growth under nutrient deprivation (61). Together, these reports highlight the complexities at work within the TME where the effects of EV-mediated signaling are likely intricately linked to feedback loops sensing the surrounding environment.

### Extracellular Vesicle–Mediated Modulation of the Tumor Immune Response

The application of rapidly evolving technologies, including single-cell RNA sequencing, to the TME has furthered our understanding of tumor-infiltrating immune cells and has exposed important underlying biology, origins, and phenotypic heterogeneity of those same cells. Due to the pivotal roles of infiltrating immune cells and their profound influence on differential responses to cancer immunotherapies, immune cell communication paradigms including EV-mediated cell–cell communication have become the focus of intense research. Exosomes serve as a signaling platform with functional implications for each state of the cancer immunity cycle, with the ability to modulate immune cells of both myeloid and lymphoid populations (62).

Within the TME, macrophages, frequently referred to as TAMs, regulate or participate in multiple fundamental processes during neoplastic pathogenesis. In addition to their well-characterized roles in clearing pathogens and cells through phagocytosis, TAMs secrete a wide array of cytokines with immunomodulatory properties that regulate key functions of both innate and adaptive immunity. In part due to their functional plasticity, TAMs have traditionally been classified as either antitumor M1 or tumor-promoting M2 populations (63). Numerous studies have reported a polarization shift in TAMs toward the protumorigenic M2 phenotype in response to exosomes released from tumor cells of ovarian, pancreatic, melanoma, colorectal, osteosarcoma, liver, and gastric origin (64). Breast cancer–derived exosomes, for example, were reported to transfer gp130 to recipient macrophages, resulting in activated STAT3 signaling and survival of the macrophage population. In addition to increasing macrophage survival, however, exosome-mediated STAT3 activation also resulted in elevated secretion of protumorigenic cytokines including interleukin (IL)-6 (65). Similarly, additional research has reported increased secretion of multiple cytokines and signaling factors including vascular endothelial growth factor A, IL-10, IL-1β, MMP-9, tumor necrosis factor alpha (TNF-α), and monocyte chemoattractant protein 1 from macrophages exposed to exosomes released from pancreatic ductal adenocarcinoma cells or liver cancer cells (66-68). While the mechanisms through which exosomes modulate TAM polarization remain unclear and are an active area of research, multiple studies have reported the polarizing and signaling effects stemming from exosome-mediated miRNA transfer. These include miR-301a-3p, miR-25-3p, miR425-5p, and miR-130b-3p, which modulate the PTEN/PI3K signaling axis; miR-222-3p, which targets SOCS3 to activate STAT3; miR-1246; andmiR-21 (69-73). There are many other studies highlighting the importance of exosome-mediated macrophage activation, and we point the reader to one of the many excellent reviews on the subject for a more in-depth analysis (4, 64, 74).

In addition to macrophages, tumor-derived exosomes have documented effects on other immune cells of myeloid origin including neutrophils (75-77), myeloid-derived suppressor cells (MDSCs) (78, 79), monocytes (72, 80-82), and dendritic cells (DCs) (83, 84). Among these, exosomes mediate many similar protumor effects on monocytes, as they represent an innate immune cell population able to differentiate further into TAMs. Monocytes, however, can also differentiate into dendritic cells, a specialized cell type with antigen-presenting functions during both innate and adaptive immunity. Interestingly, exosomes have been reported to facilitate DC-mediated immunity in part by engaging the cytosolic dsDNA sensor cGAS (85-87). In vitro, exosomes released from mouse breast cancer cells transferred dsDNA to recipient DCs, which activated cGAS, led to production of the second messenger cGAMP, and resulted in the activation of type I interferon in a STING-dependent manner (86). In vivo, tumor-derived exosomes activated the CD8^+^ T cell response, leading to the release of T cell exosomes containing both genomic and mitochondrial dsDNA. These EVs, upon interaction with DCs, triggered expression of interferon-regulated genes as a result of the DNA cargo, engaging the cGAS/STING pathway (87). MDSCs, as their name suggests, function to foster the immunosuppressive nature of the TME, and multiple reports have highlighted distinct mechanisms through which tumor-derived exosomes can enhance this function of MDSCs. Intriguingly, one such mechanism is the activation of JAK/STAT signaling in MDSCs as a result of exosomal miR-9 and miR-181a, which targeted SOCS3, in a pathway similar to that which led to TAM polarization (88). STAT3 activation has also been reported in response to renal cancer exosomes, which engaged TLR2 and enhanced MDSC suppressive functions (89). Distinctly, in a model for oral squamous cell carcinoma, exosomes released by hypoxic tumor cells activated a miR-21/PTEN/PD-L1 axis to engage the immunosuppressive effects of MDSCs on T cells, an effect that could be blocked by the simultaneous targeting of PD-L1 and miR-2 (90).

Like cells of the myeloid lineage, exosomes are similarly capable of modulating lymphoid-derived components [including natural killer (NK) cells, B lymphocytes, and T lymphocytes] of the TME, with the primary effect being the generation of an immunosuppressive and protumorigenic environment. In a glioblastoma multiforme model, for example, miR-387a delivered to NK cells by tumor exosomes downregulated expression of granzyme B, thereby impairing the cytolytic function of recipient NK cells (91). Paradoxically, and possibly highlighting the importance of the complete cargo carried by EVs rather than individual components, exosomes shed from Hsp-70^+^ colon or pancreatic tumor cells enhanced the migratory and lytic capacity of NK cells relative to exosomes released from the counterpart lines lacking Hsp-70 expression (92).

The role of B cells within the TME has gained increasing attention, and with that, our understanding of the heterogeneous subtypes and their multitude of functions has similarly advanced. In the context of cancer, B lymphocytes have numerous documented roles including immunoglobulin production, cytokine production, T cell modulation, and antigen processing (93). It is no surprise, then, that B cell responses to tumor-derived EVs are similarly varied. Several reports have suggested that exosomes can function as surveilling decoys, protecting tumor cells from systemic treatments by titrating those treatments out of circulation. B cell lymphomas can, for example, release abundant CD20 containing exosomes that reduce the efficacy of rituximab therapy. Rituximab, a monoclonal antibody treatment targeting CD20, bound to exosomes in vitro and in vivo, where it fixed complement, and ultimately this combination resulted in the consumption of complement in a manner that impeded the efficacy of humoral immunotherapy (94). Similarly, a more recent report highlighted the effect of tumor-antigen-containing exosomes shed from pancreatic ductal adenocarcinoma cells, which functioned to induce autoantibodies and reduce complement-mediated antitumor cytotoxicity (95). Furthermore, EVs of multiple tumor origins can induce the expansion of a regulatory B cell population that expresses T cell immunoglobulin and mucin-domain containing 1 (TIM-1). Mechanistically, this stems from exosomes engaging TLR2/4 through high mobility group box 1 (HMGB1) to activate the MAPK pathway in B cells and drive naive B cells to the regulatory phenotype (96). Using a glioblastoma model, researchers have also reported that the release of placenta growth factor containing EVs can convert naive CD19^+^ B cells to TGFβ-producing regulatory B cells. Intriguingly, this effect was restricted to tumor-infiltrating B cells and was not detected in peripheral B cell populations (97). It is also worth noting that researchers have reported that subcapsular sinus macrophages resident within tumor-draining lymph nodes physically block the dissemination of melanoma cell–derived EVs. Loss of this macrophage barrier due to tumor progression or in response to treatment permits interaction of those EVs with B cells and leads to the development of tumor-promoting humoral immunity (98).

Beyond macrophages, discussed previously, the most well-studied immune cell population in response to tumor EV communication is the T lymphocyte. Cytotoxic CD8^+^ T lymphocytes together with CD4^+^ T helper 1 cells are the key regulators of antitumor immunity (note that regulatory CD4^+^ T cells known as Tregs are a potent immunosuppressive subset of CD4^+^ T cells also found in the TME) (93). A growing body of evidence has demonstrated that within the TME, EVs represent a potent means to elicit a range of functional responses in T cells. Fas ligand (FasL) has been identified on tumor EVs from a multitude of sources and has been reported to induce apoptosis of T cells upon binding (99, 100). Similarly, TNF-related apoptosis-inducing ligand (TRAIL) found decorating the surface of colorectal cancer–derived EVs led to apoptotic induction in T cells in a TRAIL- and FasL-dependent mechanism (101). Perhaps the most well-known mechanism to evade the antitumor immune response is mediated by the interaction of programmed cell death-ligand 1 (PD-L1), found on tumor and TME cells, with the cognate receptor, programmed cell death protein 1 (PD-1), expressed on activated T cells. Numerous reports have emerged demonstrating both an in vitro and an in vivo response to PD-L1-decorated EVs including systematic suppression of antitumor immunity, increased tumor growth, conversion from PD-L1-negative to PD-L1-positive status, inhibition of cell killing and cell signaling in T cells, suppressed T cell proliferation, and reduced T helper 1 cell cytokine secretion (102-105).

### Tumor Extracellular Vesicles Facilitate Cell Migration and Matrix Invasion

During tumor development and progression, the ECM undergoes an extensive and dynamic reorganization. This abnormally restructured, cancerized ECM alters cancer progression by supporting cell proliferation and survival and promoting cell migration, where degraded matrix fragments, sometimes referred to as matrikines, can modulate signaling cascades through interactions with cell-surface receptors. The release of functional EVs represents a potent means through which tumor cells can facilitate ECM remodeling through the degradation of matrix proteins, reprograming of recipient cells, and/or the conditioning of a premetastatic niche.

It is now well understood that invading tumor cells will alter their behavior in response to cues received from the extracellular environment. This plasticity is in part modulated by the same families of small GTPases that regulate the pinching and release of TMVs from the cell surface including the mutually antagonistic activation/inactivation of both RhoA and Rac1. In actively invading tumor cells, RhoA activation in response to decreasing matrix rigidity causes a shift to a more amoeboid morphology characterized by increased cell rounding and an increase in TMV shedding (26). Additionally, ezrin phosphorylation at threonine-567 occurring downstream of RhoA/ROCK signaling has been linked to the formation of a stable ternary complex at the plasma membrane with the transmembrane glycoprotein podocalyxin (25). In both cases, GTPase signaling pathways converge, resulting in the release of abundant protease-loaded invasive TMVs. Once released from the cell surface, these TMVs are fully capable of degrading ECM, and this degradative activity is critical for amoeboid cell invasion (27). The predominant driver of TMV-mediated invasive capacity is MT1-MMP, which is critical for tumor cell invasion in collagen-rich environments (106). In a melanoma cell model, depleting MT1-MMP from shed TMVs by blocking protein trafficking resulted in a significant reduction in invasive capacity and a loss of directionality in amoeboid-type cells despite no change to overall TMV shedding and no alterations to other TMV cargo (30).

Increasing evidence suggests that, similar to TMVs, the shift toward an amoeboid morphology results in increased shedding of large oncosomes. Like TMVs, LOs are known to contain soluble proteases including MMP2 and MMP9, which remain bioactive within the shed oncosomes (107). Prostate cancer–derived LOs have long been known to stimulate migration of dermal and tumor endothelial cells; however, more recently they were reported to increase adhesion and invasion of recipient cells via the integrin αV–mediated activation of AKT and, intriguingly, increased MMP transcription (107, 108). Additionally, using a xenograft model system, these researchers demonstrated that the release of integrin αV–positive LOs correlated with aggressive tumor growth and, moreover, that treatment of control tumor cells with aggressive LOs would confer increased engraftment and tumor development to recipient cells that normally do not engraft in the absence of Matrigel support (108). The physical scale of most EV populations makes their identification in pathology samples difficult or impossible; however, the uncharacteristically large size of LOs means that, in addition to bodily fluids, they have repeatedly been identified in immunohistochemical analysis of human samples (107-110).

Studies have also implicated exosome secretion in directional motility and invasion of tumor cells. In this regard, invadopodia, which are invasive structures formed at the adherent cell surface, are thought to serve as docking sites for MVBs, thereby facilitating release of protease-containing exosomes (111). Furthermore, knockdown of Rab27a, a regulator of MVB biogenesis, led to loss of directionality and migration defects in fibrosarcoma and squamous carcinoma cells (112). Live confocal imaging of fibrosarcoma cells on nanopatterned dishes revealed that exosomes were being secreted at the leading edge of migrating cells (113). Recently, additional research showed that sEVs secreted from hypoxic breast cancer cell lines were shown to induce mitochondrial dynamics and integrin-linked kinase (ILK)–Akt kinase-dependent migration of normal mammary epithelial cells (114).

In recent years, reports have emerged of a large EV population, referred to as the migrasome, which is formed at the tips or intersections of retraction fibers at the trailing end of migrating cells (115-117). Migrasomes share some physical and behavioral characteristics with exosomes and microvesicles in that they contain tetraspanins and cytoskeletal proteins and shed vesicles can be taken up by neighboring cells. Notably, migrasome generation depends on cell migration. However, the mechanisms underlying migrasome biogenesis remain to be determined, and it would be of interest if they were formed by pathways similar to those described for tumor microvesicles and/or exosomes.

The literature also reports that EVs produced by cancer cells can help promote the development of metastases by altering signaling pathways in recipient cells to promote an epithelial-to-mesenchymal transition (EMT) or by conditioning a premetastatic niche. For instance, Chinese hamster ovary cells treated with EVs from aggressive U87-MG glioblastoma cells migrate more rapidly, compared with cultures of untreated cells (118). Similarly, treating urothelial cells with exosomes isolated from bladder cancer cells reduced expression of E-cadherin and β-catenin that occurs during an EMT and also enhanced cell migration (119). It is becoming more apparent with additional research that EVs of multiple types can function to condition a premetastatic niche. This includes the transfer of bioactive cargo, immune cell recruitment, and organotropism. Migration inhibitory factor contained within pancreatic cancer exosomes primes the liver for metastasis development, while MET-containing melanoma exosomes educate bone marrow progenitors, driving them toward a prometastatic phenotype (120, 121). Moreover, it was recently reported that Lin28B-high breast cancer cells establish an immunosuppressive premetastatic niche via the release of exosomes with low levels of let-7s (122). Interestingly, tumor EV-derived integrin αV was identified as the cargo responsible for the formation of a premetastatic niche that facilitates breast cancer colonization in bone (123). This study is particularly intriguing in light of prior reports that EV integrin cargo facilitates organotropism during tumor metastasis (124).

## FINDING THE NEEDLE IN THE HAYSTACK—EXTRACELLULAR VESICLE–BASED LIQUID BIOPSIES

More and more, cancers are being molecularly profiled upon diagnosis in an effort to aid in treatment design and decision-making processes. Despite the dynamic nature of the disease, procedural limitations, and lack of access to specimens, pathological examination of tissue biopsies remains the standard of care for solid tumor diagnostics. Extracellular vesicles contain a multitude of biomarkers that, if thoughtfully developed as platforms for liquid biopsies, may represent a paradigm shift in our ability to rapidly diagnose and personalize therapeutic approaches for cancer patients. This potential has led not only to expansive in vitro research but also to the establishment of many clinical trials investigating the diagnostic potential of EVs (Table 2 and Supplemental Table). While the precise meaning of the term “liquid biopsy” varies in the medical and scientific literature, the term is used broadly to encompass the collection of a bodily fluid to test for the presence of relevant biomarkers and inform patient care. Liquid biopsies can overcome many of the limitations presented by traditional surgical resection due to many factors including their accessibility in peripheral bodily fluids, their minimally invasive nature, the ability to longitudinally monitor disease, increased patient compliance, and reduced sampling bias. Currently, the main thrust of development for liquid biopsies has branched into three main fields: circulating tumor DNA (ctDNA), circulating tumor cells, and EVs, where the vast majority of research has focused on the development of exosome-based technologies.

Though not without their challenges, discussed in greater detail below, EV-based biomarkers possess multiple attributes that have heightened interest in their development. Extracellular vesicles can be isolated from virtually all peripheral bodily fluids or secretions including blood, breast milk, saliva, stool and urine, tears, amniotic fluid, pleural effusion, synovial fluid, cerebrospinal fluid (CSF), seminal and vaginal fluid, and ascites. Furthermore, because they are released from living cells, EVs contain information about active tumor cells; they are a renewing source of information to monitor disease; they are abundant, with current estimates centering at ~1 billion exosomes/mL of blood; and their extracellular organelle nature means they are comparatively stable in most physiological conditions. Additionally, as mentioned previously, EVs contain a complex mixture of molecular cargo, and their composition closely reflects the physiological state of the shedding cell and the biogenesis pathways responsible for their release. As such, by sampling EVs from peripheral fluids it is possible to more comprehensively assess the pathophysiological changes associated with disease, examine the heterogeneous changes associated with disease, and provide nearly real-time information regarding diagnosis and longitudinal monitoring of treatment response. Thus, the sampling of EVs provides an opportunity relative to other biomarkers to both isolate and enrich a tumor signature that captures multiple biological components in a single platform.

As mentioned previously, numerous studies have shown that cancer-related cargoes including proteins and nucleic acids are differentially expressed in tumors of different origins, and, as such, their loading into EVs can potentially be used to identify and distinguish cancer types. Much of the promising early work on EV biomarker development focused on the inclusion of previously characterized biomarkers within EVs and the identification of novel protein cargo, which could be exploited for further development. This includes differential inclusion of the canonical exosome marker CD63, which was elevated in ovarian cancer exosomes but minimally included in lung cancer specimens (125). CA-125 was found on ascites-derived TMVs while absent from EVs in a patient later diagnosed with benign disease. It was also enriched on serum-derived EVs relative to unfractionated serum (30). When compared with healthy controls, glypican-1 was significantly increased in serum exosomes inpatients with pancreatic cancer, and research suggested it could be used for early detection of the disease with very high diagnostic sensitivity and specificity (126). Similar studies using serum-derived T cell exosomes suggest that T cell exosomal levels of PD-1 and CD28 could function as biomarkers that can be used to monitor clinical response of metastatic melanoma patients to ipilimumab (127). More recently, researchers conducted a thorough proteomics-based investigation of plasma-derived exosomes, which revealed that these EVs could be used to identify specific cancer types and to distinguish between tumor sources with ≥90% sensitivity and specificity. The researchers found 51 specific proteins in pancreatic cancer plasma–derived exosomes and 19 specific proteins in lung cancer plasma–derived exosomes by comparing paired tumor and adjacent tissue. They were able to then expand their analysis of tumor-specific EV proteins unique to the plasma of cancer patients, which allowed them to determine that these proteins were derived not only from the TME but from distant organs and the immune system (39). These results reaffirmed the significant potential in the development of robust EV-based biomarkers.

Protein cargo is not the only cargo component that is differentially contained within tumor EVs. While the state of the field remains unsettled, there is ample evidence, as described above, for the inclusion of nucleic acid cargo, which could be used as a cancer-specific biomarker. The RNA content of circulating EVs including mRNA, noncoding RNA, lncRNA, snRNA, rRNA, tRNA, miRNA, circulating RNA, and piwi-interacting RNA is found both externally associated with isolated EVs and within the limiting membrane, where it is protected from enzymatic degradation. This increased stability, coupled with the inclusion of surface proteins such as CD47 to limit plasma clearance, likely aids in recovery and analysis for nucleic acid biomarkers (128). In addition to protein transfer via sEVs, mRNA encoding epidermal growth factor receptor variant III (EGFRvIII), the oncogenic, tumor-specific truncation of EGFR, can be recovered from serum exosomes of glioblastoma patients (129). Similarly, mutant isocitrate dehydrogenase 1 (IDH1) mRNA transcripts could be detected by digital polymerase chain reaction in CSF exosomes isolated from patients bearing IDH1 mutant glioblastoma tumors (130). Clinical data revealed that elevated expression of miR-1247-3p on serum exosomes of hepatocellular carcinoma patients correlated with progression and development of lung metastases, while exosomal miR-15a-5p was 7- to 19-fold higher in endometrial cancer patients (131, 132). Numerous additional reports have sought to examine EV RNA in the context of tumor detection, resulting in the identification of single targets or panels that are elevated in the disease context. These include lncRNAs RP11-77G23.5 and PHEX-AS1 or miR-21, mir-378, miR-139, and mir-200 in lung-cancer-patient exosomes; miR-4732-5p in ovarian cancer; mir-375, miR-501-3p, mir-574-3p, and prostate-specific antigen mRNA in prostate cancer; mir-10b in pancreatic cancer; miR-375, miR-200c, and miR-222 in breast cancer exosomes; miR-15b-3p and lncRNA HOTTIP in gastric cancer; and miR-92b, miR-21, or a separate study looking at a panel of 10 miRNA markers in hepatocellular carcinoma (133-145).

Among the most tantalizing prospects of EV-based liquid biopsy development is the ability to detect and monitor nucleic acid sequence mutations carried as vesicle cargo. Whether through RNA or DNA, mutation detection can potentially inform treatment decisions for a multitude of diseases. Combining exosomal RNA (exoRNA) with ctDNA resulted in significantly increased sensitivity, with detection of nearly 10 times more copies of mutant EGFR than ctDNA alone (146). In a similar study, researchers determined that target length (~200 bp) using a combined exoRNA and exoDNA resulted in greater mutation detection for EGFR mutations in NSCLC patients than either exoDNA alone or cell-free DNA (cfDNA) (147). In addition to exosomes, LEVs and large oncosomes can serve as ideal surrogates for development in liquid biopsy, given their ability to capture the heterogeneity of the existing tumor and the previously documented similarity to the DNA content of the originating cell (45). Follow-up studies found that lymphatic fluid contained abundant LEVs, with 50% alignment of BRAF mutational status in matched tissues (148).

While EVs hold great promise as tumor biomarkers given their ability to provide a readily accessible snapshot of cellular physiology, components, and genomic modifications, significant challenges to their development and widespread application remain. Many of these challenges stem from the dissonant methods of isolation, purification, and characterization leading to likely sampling bias toward a given EV type or subtype. When coupled with the high levels of intrinsic heterogeneity existing within biofluid-derived EVs, this results in greater limitations of sensitivity, the need for larger sample volumes to reach detection thresholds, and difficulty overcoming the low ratio of signal (tumor EV) to noise (nontumor EV). Much of the research heralding the identification of putative biomarkers also relies on procedures that are costly, time consuming, and not readily scalable for use as true diagnostics. Considerable research continues to be directed toward the development of rapid, sensitive isolation techniques to obtain EVs from clinical samples with sufficient yield and purity. As was recently demonstrated with the isolation of urinary exosomes using chimeric nanocomposites of lactoferrin conjugated 2,2-*bis* (methylol)propionic acid dendrimer-modified magnetic particles, it is possible to rapidly and efficiently isolate EVs for use in downstream analysis. The researchers were able to subsequently examine protein content and identified both up- and downregulated exosomal miRNA in the urine of prostate cancer patients relative to healthy controls (22).

## PERSPECTIVES AND FUTURE DIRECTIONS

With the increasing incidence and prevalence of cancers, the need for novel biomarker and diagnostic development is immediate and must adapt with our growing understanding of the complexity of cancer as a category of disease. Despite the pivotal biological roles for EVs during normal and pathological states, and their ability to more completely capture the dynamic heterogeneity of cancer, limited knowledge and technical challenges have stymied their clinical translation. Even with these challenges, however, EVs paired with increasingly powerful nanosensing technologies offer myriad benefits that cannot be ignored. While we have attempted to describe the current state of the field by categorizing EVs and their associated cargo, this is not meant to imply that each should function without the other when considering EV-based technologies. Each analyte proposed or used for liquid biopsy, whether it be an exosome, TMV,LO, cfDNA, or other particle, has its own advantages and disadvantages, and the research described above highlights how they can each be paired to overcome the others’ limitations.

Screening tests such as liquid biopsies are often judged by their ability to detect disease with high levels of sensitivity and specificity. However, the success of liquid biopsy and biomarker development is inextricably linked to therapeutic success; thus, even an incremental advance in our ability to detect disease is a success (149). Many of the patients who die each year from cancer do so because the disease was detected too late for surgical intervention or current first-line therapies to be effective. One mechanism to overcome this challenge is to continue to develop reliable tests that can be utilized as part of routine care to detect disease at early stages.

The overwhelming majority of research into EVs to date has been conducted on bulk isolates, overlooking their intrinsic heterogeneity. As our understanding of EV cargo has continued to develop, it becomes less and less likely that the biological effects of EVs result from the sum total of their constitutive parts. Rather, specific subtypes, or even specific cargo molecules such as mutation-containing genomic DNA, lead to a given effect. Understanding the role of specific cargo, the distinct modes of biogenesis, and the technology necessary to isolate certain EV groups will aid not only in our understanding of EV biology but also in our ability to develop novel detection assays and to reverse engineer EVs to exploit their intrinsic capabilities for good (23, 128, 150). Despite numerous challenges, the promise of EV biology and EV-based technology is enormous, and the future remains bright.

## Supplementary Material

D'Souza-Schorey Supplemental

## Figures and Tables

**Table 1:** Types of extracellular vesicles and particles (EVPs)

Paticle type	Mode of biogenesis	Name	Also known as	Lipid encapuslated?	Origin
Extracellular vesicle (EV)	Inward invagination into multivesicular body	Exosome	Classical and nonclassical exosomes Small EVs (sEVs) Heavy and light exosomes	Yes	Multivesicular endosome
	Outward budding and pinching	Arrestin domain containing protein-1 mediated microvesicle	ARMM	Yes	Plasma membrane
	Outward budding and pinching	Small microvesicles	Small EVs (sEVs)	Yes	Plasma membrane
	Outward budding and pinching	Microvesicles	Classical microvesicles Tumor-derived microvesicles (TMVs) Large EVs (LEVs) Oncosomes	Yes	Plasma membrane
	Trailing edge retraction	Migrasome	Not applicable	Yes	Plasma membrane?
	Outward budding and pinching	Large oncosome	LO	Yes	Plasma membrane
	Autophagy pathways	Secretory autophagosome	Autophagic EV	Yes	Autophagosome
	Autophagy pathways, neuronal proteostasis	Exopher	Not applicable	Yes	Plasma membrane
	Random blebbing of apoptotic cell	Apoptotic bodies	Apoptotic bleb Apoptotic EV	Yes	Plasma membrane
Extracellular particle (EP)	Unknown	Supramolecular attack particles	SMAP	No	Cytotoxic T lymphocyte dense secretory granules
	Unknown	Exomere	Not applicable	No	Unknown
	Unknown	Supermere	Not applicable	No	Unknown
	Autophagy-dependent trafficking of cytosolic DNA	Nucleosome	Extracellular chromatin particles	No	Multivesicular endosome

EVPs are a heterogeneous pool made up of multiple populations of both membrane-bound vesicles and the more recently described particles, which lack a defined lipid membrane.

**Table 2:** Summary of clinical trials investigating the use of extracellular vesicles for diagnostics

Disease	Study types	Study phases	Number of studies
Multicancer	Interventional and observational	1, 2, and 3	21
Lung cancer	Interventional and observational	1, 2, and 3	18
Melanoma	Interventional	1	2
Ovarian cancer	Interventional and observational	1 and 2	4
Hematologic cancers	Interventional and observational	1 and 2	8
Prostate cancer	Interventional and observational	1, 2, 3, and 4	51
Colorectal cancer	Interventional and observational	1	7
Hepatocellular carcinoma	Interventional and observational	Not applicable	5
Noncancer	Interventional and observational	1, 2, 3, and 4	10
Breast cancer	Interventional and observational	1, 2, and 3	16
Malignant glioma	Interventional and observational	1 and 2	5
Gastric cancer	Observational	Not applicable	2
Renal cancer	Observational	Not applicable	3
Esophageal cancer	Observational	Not applicable	2
Sarcoma	Observational	Not applicable	1
Bladder	Observational	Not applicable	1
Thyroid	Observational	Not applicable	1
Pancreatic cancer	Interventional and observational	1, 2, 3, and 4	11
Retinoblastoma	Observational	Not applicable	1

A more detailed list of studies can be found in the Supplemental Table.
