# Supplementary material for: Tumor-Derived Extracellular Vesicles: Multifunctional Entities in the Tumor Microenvironment
Source: Annu Rev Pathol. Author manuscript; Available in PMC 2023 Aug 9. (PMC10410237; doi:10.1146/annurev-pathmechdis-031521-022116)
Supplement: D'Souza-Schorey Supplemental [file NIHMS1913596-supplement-1.pdf]

NCT Number	Title	Conditions	Phases	Study Type
NCT00004847	Diagnosis of Pheochromocytoma	Pheochromocytoma;Endocrine Disease;Endocrine Diseases	Phase 1	Interventional
NCT00004878	Donor Lymphocytes to Prevent Graft-Versus-Host Disease in Patients With Chronic Myeloid Leukemia	Graft Versus Host Disease;Leukemia	Phase 2	Interventional
NCT00006241	Peripheral Stem Cell Transplantation in Treating Patients With Relapsed Low- or Intermediate-Grade Non-Hodgkin's Lymphoma	Lymphoma	Phase 2	Interventional
NCT00216060	Risedronate to Prevent Skeletal Related Events in Patients With Metastatic Prostate Cancer Commencing Hormonal Therapy	Metastatic Prostate Cancer	Phase 3	Interventional
NCT00216372	Efficacy and Safety of Lanreotide Microparticles as Palliative Treatment in Peritoneal Carcinomatosis	Intestinal Obstruction;Carcinoma;Peritoneal Neoplasms	Phase 3	Interventional
NCT00336258	US-Doppler and Procoagulant Microparticles for the Diagnosis of Asymptomatic DVT in Advanced Cancer Patients With Poor Performance Status	Deep Vein Thrombosis;Cancer		Observational
NCT00363311	Assessment Of Dutasteride (AVODART) In Extending The Time To Progression Of Low-Risk, Localized Prostate Cancer In Men	Neoplasms, Prostate	Phase 4	Interventional
NCT00429338	3T Endorectal Magnetic Resonance Spectroscopic Imaging (MRSI) of Prostate	Prostate Cancer	Not Applicable	Interventional
NCT00438464	Finasteride in Treating Patients With Stage II Prostate Cancer Who Are Undergoing Surgery	Adenocarcinoma of the Prostate;Stage II Prostate Cancer	Phase 2	Interventional
NCT00454935	Correlation Between SV2A Expression in Tumour Tissue and Efficacy of Levetiracetam in Glioma Patients With Epilepsy	Epilepsy;Glioma		Observational
NCT00520546	Clinical Value of FEC-PET Combined With Endorectal MRI for Pre-therapeutic Staging of Prostate Cancer	Prostate Cancer	Phase 3	Interventional

Supplemental Material: Annu. Rev. Pathol. Mech. Dis. 2023. 18:205-229
https://doi.org/10.1146/annurev-pathmechdis-031521-022116
Tumor-Derived Extracellular Vesicles: Multifunctional Entities in the Tumor Microenvironment
Clancy and D’Souza-Schorey

NCT00578240	Molecular Studies and Clinical Correlations in Human Prostatic Disease	Prostate Cancer		Observational
NCT00627796	Lanreotide Autogel-120 mg as First-Line Treatment of Acromegaly	Acromegaly	Phase 4	Interventional
NCT00677781	Impact of Microparticles on Postoperative Complications in Surgical Patients	Neoplasm, Hepatic;Pancreatic Neoplasms;Colorectal Neoplasms		Observational
NCT00773656	Patients Overexposed for a Prostate Adenocarcinoma	Prostate Adenocarcinoma		Observational
NCT00908960	Enoxaparin Thromboprophylaxis in Cancer Patients With Elevated Tissue Factor Bearing Microparticles	Advanced Pancreatic, Colon, Lung, Gastric and Ovarian Cancer	Phase 2	Interventional
NCT00924937	COronary Diet Intervention With Olive Oil and Cardiovascular PREVention	Myocardial Infarction;Unstable Angina;Malignancy;Cognitive Decline;Diabetes Mellitus;Metabolic Syndrome	Not Applicable	Interventional
NCT01040624	Docetaxel, Androgen Deprivation and Proton Therapy for High Risk Prostate Cancer	Prostate Cancer	Not Applicable	Interventional
NCT01285817	A Clinical Trial Using Metronomic Oral Low-dose Cyclophosphamide Alternating With Low-dose Oral Methotrexate With Continuous Celecoxib and Weekly Vinblastine in Children and Adolescents With Relapsed or Progressing soliD Tumours.	Solid Tumours.	Phase 2	Interventional
NCT01288261	Paclitaxel and Bavituximab in Treating Patients With HER2-Negative Metastatic Breast Cancer	Human Epidermal Growth Factor 2 Negative Carcinoma of Breast;Male Breast Cancer;Recurrent Breast Cancer;Stage IIIC Breast Cancer;Stage IV Breast Cancer	Phase 1	Interventional
NCT01299038	Rosuvastatin to Lower Circulating Tissue Factor Bearing Microparticles in Metastatic Breast Cancer	Breast Cancer	Phase 2	Interventional
NCT01303341	Riluzole and Sorafenib Tosylate in Treating Patients With Advanced Solid Tumors or Melanoma	Advanced Malignant Solid Neoplasm;Recurrent Melanoma;Refractory Malignant Solid Neoplasm;Stage III Cutaneous Melanoma AJCC v7;Stage IIIA Cutaneous Melanoma AJCC v7;Stage IIIB Cutaneous Melanoma AJCC v7;Stage IIIC Cutaneous Melanoma AJCC v7;Stage IV Cutaneous Melanoma AJCC v6 and v7	Phase 1	Interventional

NCT01344109	A Pilot Study of Tumor-Derived Exosomes as Diagnostic and Prognostic Markers in Breast Cancer Patients Receiving Neoadjuvant Chemotherapy	Breast Neoplasms		Observational
NCT01499381	EMBRACE1: Prostate Biorepository	Prostate Cancer		Observational
NCT01499394	Caris Biorepository Research Protocol	Cancer		Observational
NCT01753713	Dovitinib in Treating Patients With Recurrent or Progressive Glioblastoma	Adult Giant Cell Glioblastoma;Adult Glioblastoma;Adult Gliosarcoma;Recurrent Adult Brain Tumor	Phase 2	Interventional
NCT01774916	Identification of Genetic and Cellular Markers Associated With Vascular Endothelial Modifications in Cutaneous Arteriovenous Malformations	Cutaneous Arteriovenous Malformations	Not Applicable	Interventional
NCT01779583	Circulating Exosomes As Potential Prognostic And Predictive Biomarkers In Advanced Gastric Cancer Patients ("EXO-PPP Study")	Gastric Cancer		Observational
NCT01794936	Pilot Study of VTI Doppler Probe Use to Identify Neurovascular Bundle During Prostate Surgery	Prostate Cancer;Nerve Sparing Prostatectomy	Not Applicable	Interventional
NCT01798134	Microparticle Enhanced Cytotoxic Transarterial Embolization Therapy in Hepatocellular Carcinoma	Hepatocellular Carcinoma	Not Applicable	Interventional
NCT01854866	Safety and Effectiveness Study of Tumor Cell-derived Microparticles to Treat Malignant Ascites and Pleural Effusion	Malignant Pleural Effusion,;Malignant Ascites	Phase 2	Interventional
NCT01991080	The Relationship Between Relaxation or Wheat Germ Juice to the Immune Indices and Quality of Life (QoL) in Colorectal Cancer Patients on Adjuvant Chemotherapy	Colon Cancer	Not Applicable	Interventional
NCT02025361	Impact of Local Anesthesia Type on Cancer Detection Rate in Transrectal Ultrasound Guided Prostate Biopsy	Adenocarcinoma of Prostate	Phase 4	Interventional
NCT02064036	Stereotactic Boost and Long-Term Androgen Deprivation for Adenocarcinoma of the Prostate	Adenocarcinoma of the Prostate	Not Applicable	Interventional

NCT02071719	Prediction of Response to Kinase Inhibitors Based on Protein Phosphorylation Profiles in Tumor Tissue From Advanced Renal Cell Cancer Patients	Renal Cell Cancer		Observational
NCT02079272	REBECCA Study (RadiothErapy for BrEast Cancer and CArdiotoxicity)	Toxicity Due to Radiotherapy;Breast Cancer;Lesion; Cardiac	Not Applicable	Interventional
NCT02095925	Cohort Study to Identify Cancer Patients at High Risk of Venous Thromboembolism	Cancer;Deep Venous Thrombosis;Pulmonary Embolism		Observational
NCT02121600	18F-FCH PET/MRI to Assess Tumor Response in Castration Resistant Prostate Cancer	Prostate Cancer	Not Applicable	Interventional
NCT02147418	Exosome Testing as a Screening Modality for Human Papillomavirus-Positive Oropharyngeal Squamous Cell Carcinoma	Oropharyngeal Cancer		Observational
NCT02161523	The Impact of Lung Cancer-derived Fibroblasts on Mast Cells Activation	Lung Cancer		Observational
NCT02189486	Prostate Cancer Upgrading Reference Set	Prostate Cancer		Observational
NCT02195232	Cancer Associated Thrombosis and Isoquercetin (CATIQ)	Thromboembolism of Vein VTE in Colorectal Cancer;Thromboembolism of Vein in Pancreatic Cancer;Thromboembolism of Vein in Non-small Cell Lung Cancer	Phase 2;Phase 3	Interventional
NCT02237612	Diffusion-Weighted MRI in Staging Patients With Localized Prostate Cancer	Adenocarcinoma of the Prostate;Stage IIA Prostate Cancer;Stage IIB Prostate Cancer	Not Applicable	Interventional
NCT02260817	Expanded Access to Diagnostic Imaging for Staging of Recurrent Prostate Cancer	Metastatic Prostate Cancer	Phase 3	Interventional
NCT02302885	Healthy Human Control Blood Collection for Validation of Biomarker Assay	Healthy		Observational
NCT02310451	Study of Molecular Mechanisms Implicated in the Pathogenesis of Melanoma. Role of Exosomes	Metastatic Melanoma	Not Applicable	Interventional
NCT02378532	The Addition of Chloroquine to Chemoradiation for Glioblastoma	Glioblastoma Multiforme	Phase 1	Interventional
NCT02393703	Interrogation of Exosome-mediated Intercellular Signaling in Patients With Pancreatic Cancer	Pancreatic Cancer;Benign Pancreatic Disease		Observational

NCT02404363	Safety and Efficacy of Clopidogrel in Locally Advanced and Metastatic Pancreatic Adenocarcinoma Treated With Chemotherapy	Locally Advanced Pancreatic Cancer;Adenocarcinoma, Pancreas;Metastatic Pancreatic Cancer	Phase 3	Interventional
NCT02464930	Evaluation of MicroRNA Expression in Blood and Cytology for Detecting Barrett's Esophagus and Associated Neoplasia	Barrett's Esophagus;Gastroesophageal Reflux;Esophageal Adenocarcinoma		Observational
NCT02507583	Antisense102: Pilot Immunotherapy for Newly Diagnosed Malignant Glioma	Malignant Glioma;Neoplasms	Phase 1	Interventional
NCT02598557	Alternative Dosing of Exemestane Before Surgery in Treating Postmenopausal Patients With Stage 0-II Estrogen Positive Breast Cancer	Stage 0 Breast Cancer AJCC v6 and v7;Stage I Breast Cancer AJCC v7;Stage IA Breast Cancer AJCC v7;Stage IB Breast Cancer AJCC v7;Stage II Breast Cancer AJCC v6 and v7;Stage IIA Breast Cancer AJCC v6 and v7;Stage IIB Breast Cancer AJCC v6 and v7	Phase 2	Interventional
NCT02604784	Study of Efficacy and Safety of Laparoscopic Intra-abdominal Chemotherapy (PIPAC) Performed in Patients With Peritoneal Carcinomatosis From Colorectal, Ovarian, Gastric Cancer and Primary Peritoneal Tumors	Peritoneal Carcinomatosis	Phase 1;Phase 2	Interventional
NCT02605512	BreAst Cancer and Cardiotoxicity Induced by RAdioTherapy: the BACCARAT Study	Breast Cancer;Cardiac Toxicity	Not Applicable	Interventional
NCT02657460	Clinical Trial of Tumor Cell-derived Microparticles Packaging Chemotherapeutic Drugs to Treat Malignant Pleural Effusion	Malignant Pleural Effusion	Phase 2	Interventional
NCT02662621	Pilot Study With the Aim to Quantify a Stress Protein in the Blood and in the Urine for the Monitoring and Early Diagnosis of Malignant Solid Tumors	Cancer	Not Applicable	Interventional
NCT02672449	Carbon Ions Boost Followed by Pelvic Photon Radiotherapy for High Risk Prostate Cancer	Adenocarcinoma of Prostate	Not Applicable	Interventional
NCT02694562	Microparticle Enhanced Cytotoxic Transarterial Embolization Therapy	Colorectal Carcinoma	Not Applicable	Interventional

NCT02702856	Clinical Validation of a Urinary Exosome Gene Signature in Men Presenting for Suspicion of Prostate Cancer	Prostate Cancer		Observational
NCT02759718	Clinical Effectiveness of Serum Chromogranin A Levels on Diagnostic of Pancreatic Neuroendocrine Tumors	Non Functioning Pancreatic Endocrine Tumor	Phase 4	Interventional
NCT02805894	NBTXR3 Nanoparticles and EBRT or EBRT With Brachytherapy in the Treatment of Prostate Adenocarcinoma	Prostate Cancer	Phase 1;Phase 2	Interventional

NCT02834013	Nivolumab and Ipilimumab in Treating Patients With Rare Tumors	Acinar Cell Carcinoma;Adenoid Cystic Carcinoma;Adrenal Cortex Carcinoma;Adrenal Gland Pheochromocytoma;Anal Canal Neuroendocrine Carcinoma;Anal Canal Undifferentiated Carcinoma;Angiosarcoma;Apocrine Neoplasm;Appendix Mucinous Adenocarcinoma;Bartholin Gland Transitional Cell Carcinoma;Basal Cell Carcinoma;Bladder Adenocarcinoma;Breast Metaplastic Carcinoma;Cervical Adenocarcinoma;Cholangiocarcinoma;Chordoma;Colorectal Squamous Cell Carcinoma;Desmoid Fibromatosis;Endometrial Transitional Cell Carcinoma;Endometrioid Adenocarcinoma;Esophageal Neuroendocrine Carcinoma;Esophageal Undifferentiated Carcinoma;Extrahepatic Bile Duct Carcinoma;Extramammary Paget Disease;Fallopian Tube Adenocarcinoma;Fallopian Tube Transitional Cell Carcinoma;Fibromyxoid Tumor;Gallbladder Carcinoma;Gastric Neuroendocrine Carcinoma;Gastric Squamous Cell Carcinoma;Gastric Undifferentiated Carcinoma;Gastrointestinal Stromal Tumor;Gestational Trophoblastic Tumor;Giant Cell Carcinoma;Human Papillomavirus-Independent Cervical Adenocarcinoma, Clear Cell-Type;Intestinal Neuroendocrine Carcinoma;Intrahepatic Cholangiocarcinoma;Lung Carcinoid Tumor;Lung Sarcomatoid Carcinoma;Major Salivary Gland Carcinoma;Malignant Odontogenic Neoplasm;Malignant Peripheral Nerve Sheath Tumor;Malignant Solid Neoplasm;Malignant Testicular Sex Cord-Stromal Tumor;Metastatic Malignant Neoplasm of Unknown Primary;Minimally Invasive Lung Adenocarcinoma;Mixed Mesodermal (Mullerian) Tumor;Mucinous Adenocarcinoma;Mucinous Cystadenocarcinoma;Nasal Cavity	Phase 2	Interventional
NCT02862366	Role of the Circulating Procoagulants Microparticles in the Hypercoagulability of MNP Ph1-	Myeloproliferative Neoplasm		Observational
NCT02862652	Role of the Microparticles and of Tissue Factor in the Pro-thrombotic Phenotype and the Thromboembolic Complications During the Acute Lymphoblastic Leukemia in Children.	Acute Lymphoblastic Leukemia	Not Applicable	Interventional

NCT02935816	Localising Occult Prostate Cancer Metastases With Advanced Imaging TEchniques	Prostate Cancer		Observational
NCT02940977	Establishment and Clinical Assessment of a Prostate Cancer (PCa) Risk Model Based on the Updated Circulating Tumor Cell (CTC) Detection Technique	Prostatic Neoplasms;Prostatic Adenoma		Observational
NCT02977468	Effects of MK-3475 (Pembrolizumab) on the Breast Tumor Microenvironment in Triple Negative Breast Cancer	Triple Negative Breast Cancer	Phase 1	Interventional
NCT02991066	Role of Microparticles in the Coagulopathy of Acute Promyelocytic Leukemia	Acute Promyelocytic Leukemia		Observational
NCT03008551	Empagliflozin vs Metformin in PCOS	Polycystic Ovary Syndrome	Phase 2;Phase 3	Interventional
NCT03031418	Clinical Evaluation of the 'ExoDx Prostate IntelliScore' (EPI)	Cancer of Prostate		Observational
NCT03032913	Diagnostic Accuracy of Circulating Tumor Cells (CTCs) and Onco-exosome Quantification in the Diagnosis of Pancreatic Cancer - PANC-CTC	Pancreatic Ductal Adenocarcinoma (PDAC)		Observational
NCT03034850	Thrombin Generation and Platelet Activation in CRS/HIPEC	Mesothelioma; Peritoneum;Pseudomyxoma Peritonei;Peritoneal Carcinomatosis		Observational
NCT03047603	Evaluation of Diagnostic Efficiency of PIVKA-II and Other Tumor Markers in HCC	Hepatocellular Carcinoma		Observational
NCT03102268	ncRNAs in Exosomes of Cholangiocarcinoma	Cholangiocarcinoma;Benign Biliary Stricture		Observational
NCT03108677	Circulating Exosome RNA in Lung Metastases of Primary High-Grade Osteosarcoma	Lung Metastases;Osteosarcoma		Observational
NCT03134820	Time of Treatment With LMWH in Cancer Patients With Thromboembolic Disease	Venous Thromboembolism;Pulmonary Thromboembolisms;Cancer		Observational
NCT03235687	Decision Impact Trial of the ExoDx Prostate (IntelliScore)	Cancer of the Prostate	Not Applicable	Interventional

NCT03236675	Detection of Either the EML4-ALK Gene Rearrangements or the T790M EGFR Mutation in the Plasma of Advanced NSCLC Patients	Carcinoma, Non-Small-Cell Lung		Observational
NCT03236688	Detection of ARv7 in the Plasma of Men With Advanced Metastatic Castrate Resistant Prostate Cancer (MCRP)	Metastatic Castrate Resistant Prostate Cancer		Observational
NCT03250078	A Pancreatic Cancer Screening Study in Hereditary High Risk Individuals	Pancreatic Neoplasms		Observational
NCT03297346	Early Detection of Cardiovascular Changes After Radiotherapy for Breast Cancer	Breast Cancer Female	Not Applicable	Interventional
NCT03317080	Dynamic Monitoring Circulating Tumor DNA in Surgical Patients With Lung Cancer	Lung Cancer		Observational
NCT03334708	A Study of Blood Based Biomarkers for Pancreas Adenocarcinoma	Pancreatic Cancer;Pancreatic Diseases;Pancreatitis;Pancreatic Cyst		Observational
NCT03339531	Optimized 2D-RT for Prostate Cancer	Prostate Cancer	Not Applicable	Interventional
NCT03339804	Neurovascular Changes Induced by Chemotherapy	Breast Cancer;Chemotherapeutic Toxicity	Not Applicable	Interventional
NCT03407963	Feasibility of Prostatic Arterial Embolization in Low-risk Patients With Unilateral Prostate Cancer Under Active Surveillance: Monocentric Pilot Study	Prostate Cancer	Not Applicable	Interventional
NCT03429244	PSMA-PET for Biopsy and Treatment Guidance in Primary Prostate Cancer	Prostate Cancer	Phase 2	Interventional
NCT03432806	A Study of Imaging, Blood, and Tissue Samples to Guide Treatment of Colon Cancer and Related Liver Tumors	Colon Cancer;Liver Tumors		Observational
NCT03460080	Diagnostic Value of AFP-L3 and PIVKA-II in HCC	Hepatocellular Carcinoma		Observational
NCT03471468	Kinetics of Microparticles Under Chemotherapy in Patients With Gastric or Pancreatic Cancer	Pancreatic Cancer;Gastric Cancer	Not Applicable	Interventional
NCT03493984	Plant Exosomes and Patients Diagnosed With Polycystic Ovary Syndrome (PCOS) 17	Polycystic Ovary Syndrome	Not Applicable	Interventional

NCT03525262	Prostate Oncologic Therapy While Ensuring Neurovascular Conservation (POTEN-C)	Prostate Cancer Adenocarcinoma	Phase 2	Interventional
NCT03532139	Pilot Study of Rosuvastatin and Enoxaparin Thromboprophylaxis Following Ovarian Cancer Surgery (O-STAT Study)	Ovarian Cancer	Phase 2	Interventional
NCT03537599	Daratumumab and Donor Lymphocyte Infusion in Treating Participants With Relapsed Acute Myeloid Leukemia After Stem Cell Transplant	Minimal Residual Disease;Recurrent Acute Myeloid Leukemia With Myelodysplasia-Related Changes;Recurrent Adult Acute Myeloid Leukemia;Recurrent Childhood Acute Myeloid Leukemia	Phase 1;Phase 2	Interventional
NCT03542253	Combined Diagnosis of CT and Exosome in Early Lung Cancer	Early Lung Cancer		Observational
NCT03576612	GMCI, Nivolumab, and Radiation Therapy in Treating Patients With Newly Diagnosed High-Grade Gliomas	Glioma, Malignant	Phase 1	Interventional
NCT03581435	A Study of Circulating Exosome Proteomics In Gallbladder Carcinoma Patients	Proteinosis;Gallbladder Carcinoma		Observational
NCT03667885	Non-Invasive Diagnostics of Small Renal Masses	Renal Cell Carcinoma;Renal Tumor		Observational
NCT03671044	A Study to Evaluate the Efficacy and Safety of Nanosomal Docetaxel Lipid Suspension in Triple Negative Breast Cancer Patients	Triple Negative Breast Cancer	Phase 3	Interventional
NCT03679819	Single-center Trial for the Validation of High-resolution Transrectal Ultrasound (Exact Imaging Scanner ExactVu) for the Detection of Prostate Cancer	Prostate Cancer		Observational
NCT03694483	Prostasomes as Diagnostic Tool for Prostate Cancer Detection	Prostate Cancer		Observational
NCT03711890	Ultra-High Resolution Optical Coherence Tomography in Detecting Micrometer Sized Early Stage Pancreatic Cancer in Participants With Pancreatic Cancer	Pancreatic Carcinoma;Pancreatic Intraductal Papillary Mucinous Neoplasm, Pancreatobiliary-Type	Not Applicable	Interventional

NCT03732768	Study of Radspherin¬Æ in Recurrent Ovarian Cancer Subjects With Peritoneal Carcinomatosis	Peritoneal Carcinomatosis;Ovarian Cancer	Phase 1	Interventional
NCT03732781	Study of Radspherin¬Æ in Colorectal Carcinoma Subjects With Peritoneal Carcinomatosis Treated With HIPEC	Peritoneal Carcinoma;Colorectal Carcinoma	Phase 1	Interventional
NCT03738319	Non-coding RNA in the Exosome of the Epithelia Ovarian Cancer	High Grade Serous Carcinoma;Ovarian Cancer;Exosomes;Prognosis;Early Diagnosis		Observational
NCT03791073	New Biomarkers in Pancreatic Cancer Using EXPEL Concept	Oncology		Observational
NCT03800121	Study of Exosomes in Monitoring Patients With Sarcoma (EXOSARC)	Sarcoma		Observational
NCT03807947	Radial Versus Femoral Access for Superselective Embolization of Hepatocellular Carcinoma	Hepatocellular Carcinoma;Hepatocellular Carcinoma Non-resectable	Not Applicable	Interventional
NCT03811600	Exosomes Implication in PD1-PD-L1 Activation in OSAS	Sleep Apnea Syndromes, Obstructive;Cancer		Observational
NCT03821909	Acquisition of Portal Venous CTCs and Exosomes From Patients With Pancreatic Cancer by EUS	Pancreatic Cancer		Observational
NCT03824275	18F-DCFPyL Positron Emission Tomography (PET)/Computed Tomography (CT) in Men With Prostate Cancer	Prostatic Neoplasms	Phase 2;Phase 3	Interventional
NCT03830619	Serum Exosomal Long Noncoding RNAs as Potential Biomarkers for Lung Cancer Diagnosis	Lung Cancer (Diagnosis)		Observational
NCT03874559	Exosomes in Rectal Cancer	Rectal Cancer		Observational
NCT03907670	Chronic Myloid Leukemic Patients Treated With Tyrosine Kinase Inhibitor	Chronic Phase Chronic Myelogenous Leukemia		Observational
NCT03911999	Exosomal microRNA in Predicting the Aggressiveness of Prostate Cancer in Chinese Patients	Prostate Cancer		Observational
NCT03957252	Validation of ClarityDX Prostate as a Reflex Test to Refine the Prediction of Clinically-significant Prostate Cancer	Prostate Cancer		Observational

NCT03969784	Microparticles in Peritoneal Carcinomatosis of Colorectal Origin	Colorectal Carcinoma	Not Applicable	Interventional
NCT03971110	A Study of Neoadjuvant Hormone Therapy in Patient With Advanced Prostate Cancer Undergoing Radical Prostatectomy.	Advanced Prostate Cancer	Phase 4	Interventional
NCT03974204	Analyses of Exosomes in the Cerebrospinal Fluid for Breast Cancer Patients With Suspicion of Leptomeningeal Metastasis.	Breast Cancer;Leptomeningeal Metastasis	Not Applicable	Interventional
NCT03985696	Exosomes and Immunotherapy in Non-Hodgkin B-cell Lymphomas	Lymphoma, B-cell, Aggressive Non-Hodgkin (B-NHL)	Not Applicable	Interventional
NCT04011865	Robot-assisted vs Laparoscopic Radical Prostatectomy for Prostate Cancer Treatment	Prostate Cancer	Not Applicable	Interventional
NCT04053855	Evaluation of Urinary Exosomes Presence From Clear Cell Renal Cell Carcinoma	Clear Cell Renal Cell Carcinoma		Observational
NCT04081194	Cell Free Circulating Nucleic Acids as New Tumor Diagnostics From Human Plasma Samples.	New Tumor Diagnostics From Human Plasma Samples		Observational
NCT04093375	Radical Prostatectomy Versus Radical Radiotherapy for Locally Advanced Prostate Cancer	Prostatic Neoplasms	Not Applicable	Interventional
NCT04100811	Identification of Clinically Insignificant or Significant Prostate Cancer With the miR Scientific Sentinel‚Ñ¢ Platform	Prostate Cancer		Observational
NCT04131231	Safety and Effectiveness of MPCD Therapy on the Treatment of Malignant Pleural Effusion	Lung Cancer;Breast Cancer;Malignant Pleural Effusion	Not Applicable	Interventional
NCT04155359	Clinical Evaluation of the miR Sentinel BCa‚Ñ¢ Test to Diagnose Bladder Cancer in Hematuria Patients	Bladder Cancer		Observational
NCT04164134	New Strategies to Detect Cancers in Carriers of Mutations in RB1	Retinoblastoma;Secondary Primary Malignancies After Retinoblastoma		Observational
NCT04182893	Clinical Study of ctDNA and Exosome Combined Detection to Identify Benign and Malignant Pulmonary Nodules	Pulmonary Nodules		Observational

NCT04225299	Evaluation of Efficacy of TOOKAD¬Æ (VTP) Versus Active Surveillance for Intermediate Risk Localized Prostate Cancer	Localized Prostate Cancer	Phase 3	Interventional
NCT04258735	Genetic Characteristics of Metastatic Breast Cancer Patients	Metastatic Breast Cancer	Not Applicable	Interventional
NCT04262154	Study of Abiraterone Acetate, Atezolizumab, GnRH Analog and Radiation Therapy in Men With Newly Diagnosed Hormone-sensitive Prostate Cancer	Metastatic Prostate Cancer	Phase 2	Interventional
NCT04288141	A Study to Measure the Expression of the HER2-HER3 Dimer in Tumour and Blood (Exosomes) Samples From Patients With HER2 Positive Breast Cancer Receiving HER2 Targeted Therapies	HER2-positive Breast Cancer		Observational
NCT04307056	Evaluation of HIFU in TREATMENT OF LOCALIZED PROSTATE CANCER and OF RECURRENCE AFTER RADIOTHERAPY	Prostate Cancer;Ultrasound Therapy	Not Applicable	Interventional
NCT04315753	Circulating and Imaging Biomarkers to Improve Lung Cancer Management and Early Detection	Lung Cancer		Observational
NCT04323579	Validation of Multiparametric Models and Circulating and Imaging Biomarkers to Improve Lung Cancer EARLY Detection.	Lung Cancer		Observational
NCT04340245	Exploiting Risk-Based Risk Stratification in Early Prostate Cancer to Discriminate Progressors From Non-Progressors	Prostate Cancer		Observational
NCT04357717	ExoDx Prostate Evaluation in Prior Negative Prostate Biopsy Setting	Prostate Cancer		Observational
NCT04394572	Identification of New Diagnostic Protein Markers for Colorectal Cancer	Colorectal Cancer		Observational
NCT04427475	Prediction of Immunotherapeutic Effect of Advanced Non-small Cell Lung Cancer	NSCLC Patients	Not Applicable	Interventional

NCT04499794	The Study of Exosome EML4-ALK Fusion in NSCLC Clinical Diagnosis and Dynamic Monitoring	Untreated Advanced NSCLC Patients;FISH Identified ALK Fusion Positive or Negative		Observational
NCT04523389	Contents of Circulating Extracellular Vesicles: Biomarkers in Colorectal Cancer Patients	Colorectal Cancer		Observational
NCT04529915	Multicenter Clinical Research for Early Diagnosis of Lung Cancer Using Blood Plasma Derived Exosome	Lung Cancer		Observational
NCT04530890	Interest of Circulating Tumor DNA in Digestive and Gynecologic/Breast Cancer	Breast Cancer;Digestive Cancer;Gynecologic Cancer;Circulating Tumor DNA;Exosomes	Not Applicable	Interventional
NCT04556916	Early Detection of Prostate Cancer	Prostate Cancer	Not Applicable	Interventional
NCT04568161	Effect of Anthracyclines and Cyclophosphamide on Cardiovascular Responses	Cardiotoxicity;Cardiovascular Disease;Neurovascular Disorder;Endothelial Disfunction;Breast Cancer	Not Applicable	Interventional
NCT04629079	Improving the Early Detection of Lung Cancer by Combining Exosomal Analysis of Hypoxia With Standard of Care Imaging	Lung Cancer		Observational
NCT04636788	Circulating Extracellular Exosomal Small RNA as Potential Biomarker for Human Pancreatic Cancer	Pancreas Adenocarcinoma	Not Applicable	Interventional
NCT04653701	Safety and Efficacy of DEB-TACE Performed With a Novel Reflux-control Microcatheter in Patients With HCC	Hepatocellular Carcinoma;Liver Cancer		Observational
NCT04661176	Evaluation of the Sentinel,Ñ¢ PCC4 Assay for Diagnosis, Prognosis and Monitoring of Prostate Cancer in Puerto Rico	Prostate Cancer		Observational
NCT04720599	Clinical Evaluation of ExoDx Prostate(IntelliScore) in Men Presenting for Initial Prostate Biopsy	Urologic Cancer		Observational
NCT04742608	Development of Liquid Biopsy Technologies for Noninvasive Cancer Diagnostics in Patients With Suspicious Thyroid Nodules or Thyroid Cancer	Thyroid Gland Carcinoma;Thyroid Gland Nodule		Observational

NCT04781062	Development of a Horizontal Data Integration Classifier for Noninvasive Early Diagnosis of Breast Cancer	Breast Cancer	Not Applicable	Interventional
NCT04870567	HDR Brachytherapy vs SABR in Early-intermediate Prostate Cancer	Biochemical Relapse Free Survival;Complications Rates (Erectile Dysfunction, GI, GU Complications)	Not Applicable	Interventional
NCT04913545	The Senstivity and Specificity of Using Salivary miRNAs in Detection of Malignant Transformation of Oral Lesions.	Oral Premalignant Lesions		Observational
NCT04939324	Molecular Profiling of Exosomes in Tumor-draining Vein of Early-staged Lung Cancer	Lung Cancer;Exosomes;Non Small Cell Lung Cancer	Not Applicable	Interventional
NCT04960956	Glycosylation of Exosomes in Prostate and Urothelial Carcinoma	Prostate Cancer;Urothelial Carcinoma		Observational
NCT05010343	Functional Image-Guided Carbon Ion Irradiation With Simultaneous Integrated Boost for Prostate Cancer	Localized Prostate Cancer	Phase 2	Interventional
NCT05038722	Function of Platelets Used for Transfusions	Thrombocytopenia;Hematologic Malignancy		Observational
NCT05092009	Lung Cancer Organoids and Patient Derived Tumor Xenografts	Lung Cancer		Observational
NCT05101655	Construction of Microfluidic Exosome Chip for Diagnosis of Lung Metastasis of Osteosarcoma	Osteosarcoma;Pulmonary Metastases		Observational
NCT05123391	Randomized Pilot Study of Radiosurgery for the Treatment of Non-metastatic Prostate Cancer	Prostatic Neoplasms;Radiosurgery;Quality of Life	Not Applicable	Interventional
NCT05131776	EUS-guided Intra-tumour Injection of OncoSil for Locally Advanced Pancreatic Carcinoma.	Pancreatic Cancer;Endoscopic Ultrasound	Phase 2;Phase 3	Interventional
NCT05141760	18F-PSMA PET and MRI in the Primary Staging of Prostate Cancer Patients	Prostate Cancer	Phase 2	Interventional
NCT05146505	miRNAs in High Grade Serous Ovarian Cancer	High Grade Serous Ovarian Cancer		Observational
NCT05159050	Intraperitoneal Paclitaxel-loaded TPM for Treatment of Peritoneal Carcinomatosis	Peritoneal Carcinomatosis	Phase 1	Interventional
NCT05191849	Circulating EV Long RNA Profiles in SCLC	Small Cell Lung Cancer	Not Applicable	Interventional

NCT05192252	Correlation Between Seminal Vesicle Size and Duration of Sexual Abstinence	Suspected Prostate Cancer		Observational
NCT05218759	Exosomes Detection for the Prediction of the Efficacy and Adverse Reactions of Anlotinib in Patients With Advanced NSCLC	Non-Small Cell Lung Cancer	Not Applicable	Interventional
